# No changes in corticospinal excitability, biochemical markers, and working memory after six weeks of high‐intensity interval training in sedentary males

**DOI:** 10.14814/phy2.14140

**Published:** 2019-06-07

**Authors:** Chiara Nicolini, Stephen Toepp, Diana Harasym, Bernadeta Michalski, Margaret Fahnestock, Martin J. Gibala, Aimee J. Nelson

**Affiliations:** ^1^ Department of Kinesiology McMaster University Hamilton Ontario Canada; ^2^ School of Biomedical Engineering McMaster University Hamilton Ontario Canada; ^3^ Department of Psychiatry & Behavioral Neurosciences McMaster University Hamilton Ontario Canada

**Keywords:** Aerobic exercise, BDNF, Cathepsin B, ICF, SICI, TMS

## Abstract

A single bout of aerobic exercise modulates corticospinal excitability, intracortical circuits, and serum biochemical markers such as brain‐derived neurotrophic factor (BDNF) and insulin‐like growth factor 1 (IGF‐1). These effects have important implications for the use of exercise in neurorehabilitation. Here, we aimed to determine whether increases in cardiorespiratory fitness (CRF) induced by 18 sessions of high‐intensity interval training (HIIT) over 6 weeks were accompanied by changes in corticospinal excitability, intracortical excitatory and inhibitory circuits, serum biochemical markers and working memory (WM) capacity in sedentary, healthy, young males. We assessed motor evoked potential (MEP) recruitment curves for the first dorsal interosseous (FDI) both at rest and during tonic contraction, intracortical facilitation (ICF), and short‐interval intracortical inhibition (SICI) using transcranial magnetic stimulation (TMS). We also examined serum levels of BDNF, IGF‐1, total and precursor (pro) cathepsin B (CTSB), as well as WM capacity. Compared to pretraining, CRF was increased and ICF reduced after the HIIT intervention, but there were no changes in corticospinal excitability, SICI, BDNF, IGF‐1, total and pro‐CTSB, and WM capacity. Further, greater CRF gains were associated with larger decreases in total and pro‐CTSB and, only in Val/Val carriers, with larger increases in SICI. Our findings confirm that HIIT is efficacious in promoting CRF and show that corticospinal excitability, biochemical markers, and WM are unchanged after 18 HIIT bouts in sedentary males. Understanding how aerobic exercise modulates M1 excitability is important in order to be able to use exercise protocols as an intervention, especially in rehabilitation following brain injuries.

## Introduction

Aerobic exercise promotes brain health and function. Indeed, exercise has been shown to improve learning and memory, delay cognitive decline, and protect against brain atrophy in healthy aging individuals (Barnes et al. 2003; Colcombe and Kramer 2003; Weuve et al. 2004; Yaffe et al. 2009; Sofi et al. 2011; Gomez‐Pinilla and Hillman 2013; Ludyga et al. 2016). Exercise programs have been also shown to reduce blood pressure, insulin resistance, and brain injury as well as to delay the onset and progression of neurodegenerative diseases such as Alzheimer's and Parkinson's (Bergen et al. 2002; Whelton et al. 2002; Cuff et al. 2003; Teri et al. 2003; Rovio et al. 2005; Crizzle and Newhouse 2006; O'Leary et al. 2006; Stevens and Killeen 2006; Rabadi 2007; van der Heijden et al. 2010; Dimeo et al. 2012; Shu et al. 2014; Chin et al. 2015). Further, exercise plays protective and therapeutic roles in depression (Blumenthal et al. 1999; Strawbridge et al. 2002; Dunn et al. 2005; Singh et al. 2005; Nabkasorn et al. 2006; Rethorst and Trivedi 2013; Kvam et al. 2016; Schuch et al. 2016). Despite the numerous human studies highlighting the importance of exercise in maintaining brain function and health, exercise‐induced functional changes in the brain and the underlying molecular mechanisms largely remain to be elucidated in humans. Changes in M1 excitability have been observed following a single session of exercise. Yamaguchi et al. (2012) have shown that short‐term, low‐intensity pedaling decreased short‐interval intracortical inhibition (SICI) in the motor cortical representation of the tibialis anterior and soleus muscles. Notably, the reduction in SICI is not restricted to the motor cortical representations of the exercising muscles. Indeed, decreased SICI has been demonstrated in M1 areas representing resting upper limb muscles such as the first dorsal interosseous (FDI) after one session of lower‐limb exercise (e.g., biking) (Takahashi et al. 2011; Singh et al. 2014a, 2014b; Lulic et al. 2017). Further, Singh et al. (2014a, 2014b) have observed increased intracortical facilitation (ICF), while Lulic et al. (2017) found decreased ICF in the M1 representations of upper limb muscles not involved in the exercise following a single biking session. Lastly, enhanced corticospinal excitability in the nonexercising FDI has been reported after a single session of moderate‐intensity lower‐limb aerobic exercise in fit but not in low‐to‐moderately fit individuals (Lulic et al. 2017). Taken together, these findings suggest that acute exercise modulates plasticity of motor cortical representations of both exercising and resting muscles. To date, however, no study has examined whether long‐term aerobic exercise training using the lower limbs induces changes in the excitability of resting upper limb muscle representations.

Animal studies have implicated the neurotrophin BDNF and the growth factor IGF‐1 in mediating the beneficial effects of exercise on hippocampal function and structure as well as cognition (Neeper et al. 1996; Oliff et al. 1998; Carro et al. 2000; Trejo et al. 2001; Gómez‐Pinilla et al. 2002; Vaynman et al. 2004; Berchtold et al. 2005; Ding et al. 2006; Huang et al. 2006; Cotman et al. 2007; Stranahan et al. 2009; Bechara and Kelly 2013). In humans, findings are less clear since exercise‐induced increases in peripheral BDNF have been consistently shown only immediately after a single bout of aerobic exercise (Gold et al. 2003; Ferris et al. 2007; Goekint et al. 2008; Tang et al. 2008; Bos et al. 2011; Cho et al. 2012; Heyman et al. 2012; Schmolesky et al. 2013; Mang et al. 2014; Skriver et al. 2014; Saucedo Marquez et al. 2015). Studies involving longer exercise interventions (i.e., 6 weeks up to 1 year) have reported mixed results. While the majority of reports have found no changes in circulating BDNF and IGF‐1 at the end of the training period (Schiffer et al. 2009; Seifert et al. 2010; Erickson et al. 2011; Ruscheweyh et al. 2011; Voss et al. 2013; Maass et al. 2016; Gourgouvelis et al. 2018), Zoladz and colleagues (2008) demonstrated increased plasma BDNF after 5 weeks of endurance training in physically active male adults. Further, Leckie et al. (2014) found that 1 year of moderate‐intensity walking significantly elevated serum BDNF only in individuals older than 65 years of age. Lastly, Heisz et al. (2017) reported that, although no group differences in serum BDNF were found following 6 weeks of high‐intensity interval training in young adults, participants with greater fitness improvements had higher serum BDNF levels than their counterparts with lower fitness gains. One of the goals of the present study was to investigate whether serum BDNF was enhanced after 6 weeks of high‐intensity interval training in sedentary males.

High‐intensity interval training (HIIT) is as efficacious a protocol at improving aerobic fitness as traditional endurance training despite the reduced time commitment (Batacan et al. 2017). Phillips et al. (2017) recently showed that training using a protocol that involved five 1‐min intervals at an intensity of ~125% peak workload (*W*_peak_; determined during a peak oxygen uptake, VO2_peak_, test) improved cardiorespiratory fitness (CRF) by ~10% in both men and women when performed three times per week for 6 weeks. In the present study, we sought to investigate whether gains in aerobic fitness induced by a similar HIIT protocol were accompanied by changes in TMS‐assessed corticospinal excitability and intracortical circuits (SICI, ICF) in sedentary, healthy males. Further, as previous studies have failed to demonstrate whether long‐term aerobic exercise increases serum BDNF and IGF‐1 (Voss et al. 2013; Leckie et al. 2014; Maass et al. 2016; Heisz et al. 2017), we also examined whether serum levels of BDNF, IGF‐1, and cathepsin B, a myokine that has been associated with CRF and memory (Moon et al. 2016), were elevated after the HIIT protocol. Lastly, since evidence suggests that exercise improves memory and executive function (Colcombe and Kramer 2003; Smith et al. 2010), we examined whether working memory (WM), which is defined as the set of cognitive skills involved in the management and manipulation of information drawn from short‐ and long‐term memory (Baddeley 1992, 2010; Engle 2002; Conway et al. 2005; Fenesi et al. 2015), was increased following 6 weeks of HIIT.

## Methods

### Subjects

Eighteen healthy males (23.1 ± 3.5 years) were recruited to participate in the study. All participants were deemed sedentary, which was defined as engaging in ≤60 min of exercise/week (Little et al. 2011; Heisz et al. 2017). Handedness was determined using a modified Edinburgh inventory questionnaire, which provides a laterality quotient (LQ) (Oldfield 1971). LQ scores range from – 100 (strong left‐hand preference) to + 100 (strong right‐hand preference) with 0 indicating ambidexterity (Oldfield 1971). In the present study, all participants were right handed (LQ: + 90.00 ± 10.99). The experimental protocol conformed to the declaration of Helsinki and was approved by the Hamilton Integrated Research Ethics Board. All participants provided informed written consent before participation.

### Pre‐ and post‐training procedures

**Figure 1:**
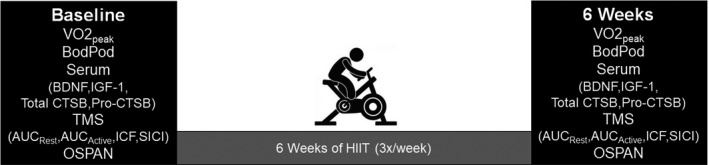
Study timeline. Cardiorespiratory fitness (VO2_peak_), body composition (BodPod), serum levels of brain‐derived neurotrophic factor (BDNF), insulin‐like growth factor 1 (IGF‐1), total cathepsin B (CTSB) and pro‐CTSB, area under the recruitment curve at rest and during tonic contraction (AUC_R__est_ and AUC_A__ctive_), intracortical facilitation (ICF), short‐interval intracortical inhibition (SICI), and WM capacity (automated operation span task, OSPAN) were assessed before (baseline) and after 6 weeks of high‐intensity interval training (HIIT).

At baseline and after the 6‐week exercise intervention, participants completed a body composition assessment, a ramp test to volitional fatigue on a cycle ergometer (Lode Excalibur v2.0, Groningen, the Netherlands) and a memory test (Fig. 1).

Fat and fat‐free mass were determined using air‐displacement plethysmography (BodPod, COSMED, Concord, California). Peak oxygen uptake (VO2_peak_) was measured using an online gas collection system (Moxus modular oxygen uptake system, AEI technologies, Pittsburgh, Pennsylvania). After a 2‐min warm‐up at 50 W, the workload was increased by 1 W every 2 sec until volitional fatigue (Little et al. 2011; Percival et al. 2015). For each participant, VO2_peak_ was calculated based on the highest value averaged over 30 sec. To assess WM capacity, participants were administered an automated operation span task (OSPAN) (Unsworth et al. 2005) using Inquisit software (millisecond, http://www.millisecond.com/download/library/OSPAN/). One math equation followed by one letter was presented, and participants were required to solve the math equations while remembering the sequence of letters. A practice session, which was comprised of three sections, preceded the actual experimental trial. During the first section of the practice session, a sequence of letters appeared on the screen and participants was required to recall the letters in the same order in which they were presented. In the second practice section, participants were presented with math equations and asked to indicate whether solutions were correct or incorrect. The third practice section included a series of math equations and letter sequences. After each math equation‐letter string, participants were required to recall the letters in the order in which they were presented. The experimental trial consisted of three to seven math equation‐letter sequence sets presented in random order. Each set was repeated three times for a total of 75 letters and 75 math problems. For each participant, the number of correct words recalled in the correct order was summed to obtain the absolute OSPAN score (Unsworth et al. 2005).

### High‐intensity interval training (HIIT)

**Figure 2:**
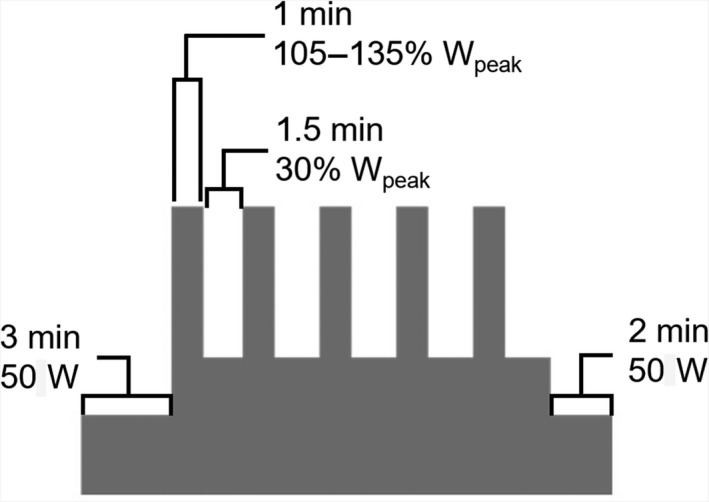
High‐Intensity interval training protocol (HIIT). A 3‐min warm‐up at 50 W was followed by five 1‐min high‐intensity bouts at ~105–135% of participants’ peak power output (*W*_peak_) with 1.5‐min recovery at 30% W_peak_ between bouts and a 2‐min cool‐down at 50 W to end. Each HIIT session was supervised, lasted 17.5 min, and was carried out on a stationary cycle ergometer.

All participants completed a supervised HIIT intervention that involved 3 sessions per week for 6 weeks. The exercise protocol was performed on an electronically braked cycle ergometer and consisted of a 3‐min warm‐up at 50 W, five 1‐min high‐intensity cycling intervals at ~105–135% of the participant's peak power output (*W*_peak_; determined during the VO2_peak_ test) interspersed with 1.5 min of active recovery at 30% *W*_peak_, and a 2‐min cooldown at 50 W (Fig. 2), for a total duration of 17.5 min (Phillips et al. 2017). Individualized workloads were determined on Visit 2, where participants were asked, after a 2‐min warm‐up at 50 W, to perform 1‐min bouts of exercise starting at 85% *W*_peak_ interspersed with 90‐sec recovery intervals at 30% *W*_peak_. Wattage was increased by 10% (e.g., 95%, 105%, etc.) until participants were unable to complete a full 1‐min interval. The workload of the last exercise interval that participants were able to complete was used as the target workload for the high‐intensity intervals in the subsequent training sessions (Phillips et al. 2017). During these sessions, if a participant was unable to complete a high‐intensity interval at the target workload, the intensity was decreased by 10% *W*_peak_ to ensure the completion of all 5 intervals. When a participant was able to complete all five high‐intensity intervals at the target workload, the target intensity was increased by 10% *W*_peak_ on the following training session.

### Electromyography (EMG)

EMG was recorded bilaterally from FDI using surface electrodes (9 mm diameter Ag–AgCl). FDI was chosen because previous studies using this muscle have reported changes in corticospinal excitability and intracortical circuits after an acute bout of lower‐limb exercise (Takahashi et al. 2011; Smith et al. 2014; Lulic et al. 2017). EMG from the FDI muscle was recorded using a monopolar electrode montage whereby one electrode was placed over the muscle belly and referenced to a second electrode positioned over the metacarpal‐phalangeal index joint. Recordings were band‐pass filtered between 20 Hz and 2.5 kHz, amplified by 1000× (Intronix Technologies Corporation Model 2024F, Bolton, Ontario, Canada) and sampled at 5 kHz (Power1401, Cambridge Electronics Design, Cambridge, UK). All participants maintained a supine position of the forearms, as corticospinal excitability depends on limb posture (Forman et al. 2016).

### Maximum voluntary contraction (MVC)

To determine MVC, each participant was asked to complete three isometric contractions of the FDI against an immovable structure as described in Lulic et al. (2017). Each contraction lasted 5 sec, and participants were given rest intervals of at least 30 sec in between contraction trials. The greatest maximum EMG activity obtained from the three trials was considered FDI MVC for a given participant. The EMG voltage corresponding to 10% MVC was calculated and displayed on the oscilloscope using a horizontal target line. Participants had to match this line when maintaining a contraction with their FDI muscle during the acquisition of active motor threshold (AMT) and active motor‐evoked potential (MEP) recruitment curve (described below).

### Maximum M‐wave (M‐Max)

M‐Max or the peak‐to‐peak amplitude of the maximum M‐wave elicited from the right FDI following stimulation of the ulnar nerve at the wrist was collected using a constant current stimulator (Digitimer, DS7AH; Welwyn Garden City, UK) and a bar electrode (cathode proximal). Square wave pulses with a 200‐*μ*sec pulse width were delivered, and stimulation intensity was increased by 1 mA until the M‐wave ceased to increase for 3 consecutive trials.

### Transcranial magnetic stimulation (TMS)

TMS was delivered to M1 using a customized 50‐mm‐diameter figure‐of‐eight branding coil connected to a Magstim Plus stimulator (Magstim, Whitland, UK). The TMS coil was positioned at a 45° angle with respect to the sagittal place to induce a posterior–anterior current. Right FDI motor hotspot was identified within the left hemisphere M1 as the cortical location that elicited the greatest and most consistent MEPs in the muscle at rest. Motor hotspot was then marked using Brainsight Neuronavigation (Rogue Research, Montreal, Canada). Resting motor threshold (RMT) and AMT for FDI were determined at the motor hotspot using the maximum‐likelihood parameter estimation by the sequential testing (ML‐PEST) method (Ah Sen et al. 2017). The freeware for ML‐PEST (TMS Motor Threshold Assessment Tool, MTAT 2.0) was obtained online (http://www.clinicalresearcher.org/software.html), and the assessment without a priori information option was used. The ML‐PEST algorithm was stopped after 20 stimuli (Ah Sen et al. 2017). During AMT acquisition, the horizontal target line on the oscilloscope provided a visual feedback to participants while they maintained a contraction with their right FDI of 10% MVC. MEP recruitment curves (RCs) were obtained in the FDI both at rest and while maintaining a contraction by delivering eight TMS pulses at intensities of 90%, 100%, 110%, 120%, 130%, 140%, 150%, 160%, 170%, 180%, 190%, 200% RMT or AMT in a randomized order (Lulic et al. 2017). For MEP RCs, the mean peak‐to‐peak MEP amplitude at each TMS intensity between 90 and 150% RMT or AMT was calculated by averaging the eight trials. Subsequently, the area under the recruitment curve (AUC) was obtained by trapezoidal integration of the resting and active RCs and normalized to M‐Max (i.e., AUC_Rest_ and AUC_Active_). ICF and SICI were assessed using paired‐pulse TMS paradigms as described in Lulic et al. (2017) with minor modifications. The conditioning stimulus (CS) was set to a TMS intensity of 90% AMT, while the test stimulus (TS) was set to evoke MEPs with peak‐to‐peak amplitudes of 1 mV in the resting FDI. CS and TS were separated by an interstimulus interval (ISI) of 10 msec (ICF) or 2 msec (SICI). For each circuit, 15 unconditioned (MEP_TS_) and conditioned (MEP_CS‐TS_) trials were randomly delivered. SICI and ICF were assessed by calculating the peak‐to‐peak amplitude of the unconditioned (MEP_TS_) and conditioned (MEP_CS‐TS_) MEP and then by computing the ratio of conditioned over unconditioned MEP (MEP_CS‐TS_/MEP_TS_). All single‐ and paired‐pulse TMS measures were collected both before (baseline) and after (6 weeks) the 6‐week HIIT training (Fig. 1).

### BDNF, IGF‐1, total Cathepsin B (CTSB), and pro‐CTSB ELISAs

Blood was drawn from 12‐h fasted participants in the morning into BD Vacutainer 10‐mL increased silica act clot activator, silicone‐coated tube (BD, Franklin Lakes, NJ, USA) before (baseline), and after (6 weeks) HIIT training (Fig. 1). Both blood draws (at baseline and after 6 weeks of HIIT) were “resting,” that is, they took place before exercise was performed. Upon collection, blood samples were allowed to clot by leaving them undisturbed at room temperature for ~45 min and then centrifuged at 3488*g* for 10 min at 4°C. Serum was aliquoted and stored at −80°C prior to use. Serum levels of BDNF, IGF‐1, total CTSB, and pro‐CTSB were measured using human BDNF DuoSet ELISA kit (DY248), human IGF‐I/IGF‐1 Quantikine ELISA kit (DG100), human total Cathepsin B DuoSet ELISA (DY2176) and human pro‐Cathepsin B DuoSet ELISA (DY953) (R&D Systems, Minneapolis, MN) according to manufacturers’ protocols. A standard curve of recombinant BDNF, IGF‐1, total CTSB, and pro‐CTSB was run on each plate. Samples and standards were run in duplicate.

### Genotyping

A 2‐mL saliva sample was collected from each participant using a DNA collection kit (Oragene•DNA OG‐500, Genotek, Ottawa, Ontario, Canada). Samples were then sent to GenoFIND Genomic Services (Norcross, Georgia) for processing. Only the region that surrounds the single‐nucleotide polymorphism (SNP) Val66Met (rs6265) on the BDNF gene was examined using a TaqMan^®^ Single Tube Assay. The genotyping results revealed that ten participants were Val/Val carriers, six Val/Met, and 2 Met/Met.

### Statistical analysis

Normality was assessed using the Shapiro–Wilks test. Data that were not normally distributed were square root transformed to meet the assumption of normality and then analyzed using parametric statistics. Data that were square root transformed are indicated in Table 1. Dependent measures included VO2_peak_, AUC_Rest_, AUC_Active,_ ICF, SICI, BDNF, IGF‐1, total CTSB, pro‐CTSB, and OSPAN absolute score and were assessed using two‐tailed paired Student's *t*‐tests. Effect sizes were calculated using Cohen's d. Hierarchical linear regression analysis was used to determine whether the percent change in CRF (VO2_peak_ %Δ) was associated with the percent change in each dependent measure and whether there was an interaction between VO2_peak_ %Δ and BDNF Vl66Met polymorphism (Brown et al. 2014). The first regression model (model 1) was corrected for age and included only the independent continuous term VO2_peak_ %Δ. The second regression model (model 2) included an interaction term between VO2_peak_ %Δ and the categorical term BDNF genotype (Val/Val, Val/Met). Significant interactions were further analyzed by stratifying participants by BDNF genotype (Val/Val, Val/Met) and by rerunning model 1 on each cohort (Val/Val, Val/Met) separately. Due to their low sample size (*n* = 2), Met/Met carriers were not included in the hierarchical linear regression analysis. Outliers, defined as values above or below 1.5× the interquartile range (IQR), were removed. Statistical significance was set at *P* < 0.05.

**Table 1:** Group‐averaged means (with SD) of measures

Measures	Baseline (*n* = 18)	6 Weeks (*n* = 18)	Paired *t*‐test and effect size
VO2_peak_	34.9 ± 4.6	39.3 ± 5.0	***P*** ** < 0.001, ** ***d*** ** = 0.894**
AUC_Rest_ Sqrt Transform	2.19 ± 0.51	2.11 ± 0.60	*P* = 0.541, *d* = 0.144
AUC_Active_	8.54 ± 3.46	8.59 ± 2.77	*P* = 0.933, *d* = 0.016
ICF	1.30 ± 0.27	1.15 ± 0.19	***P*** ** = 0.048, ** *d* = **0.656**
SICI	0.52 ± 0.25	0.50 ± 0.20	*P* = 0.752, *d* = 0.088
BDNF Sqrt Transform	4.21 ± 1.06	4.37 ± 0.95	*P* = 0.549, *d* = 0.159
IGF‐1	160.90 ± 41.15	154.50 ± 35.10	*P* = 0.198, *d* = 0.167
Total CTSB	30.32 ± 12.17	31.77 ± 11.85	*P* = 0.133, *d* = 0.121
Pro‐CTSB	15.39 ± 6.45	15.93 ± 6.72	*P* = 0.391, *d* = 0.082
OSPAN	49.67 ± 13.51	53.11 ± 11.57	*P* = 0.142, *d* = 0.274

AUC, area under the curve; BDNF, brain‐derived neurotrophic factor; CTSB, cathepsin B; ICF, intracortical facilitation; IGF‐1, insulin‐like growth factor 1; OSPAN, operation span task; SICI, short‐interval intracortical inhibition; VO2_peak_, peak oxygen uptake. Bold font indicates statistical significance.

## Results

All participants successfully completed the experiment. Group means and statistical analyses are reported in Table 1.

**Figure 3:**
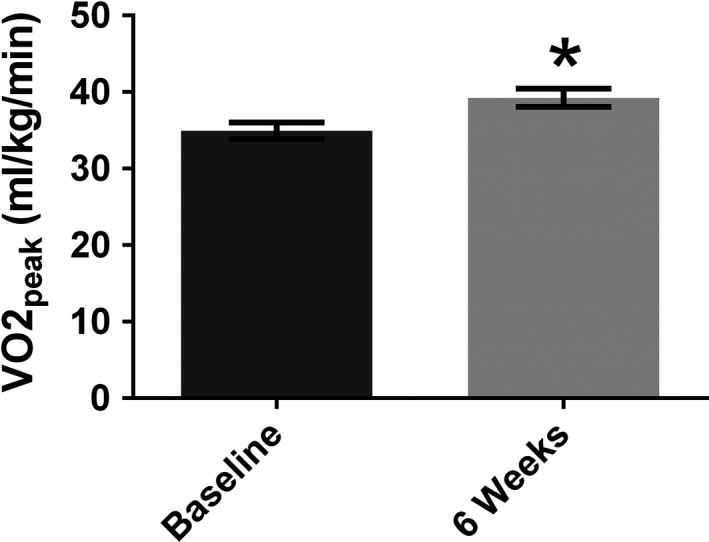
Cardiorespiratory fitness. Group‐averaged VO2_peak_ (with standard error) for all participants (*n* = 18), showing that low‐volume 5‐by‐1 HIIT significantly increased aerobic capacity in sedentary males after 6 weeks. * indicates significance of *P* < 0.05.

The supervised, 6‐week, 5‐by‐1‐min HIIT protocol resulted in an overall robust increase (~12%) in participants’ CRF (VO_2peak_) (Fig. 3, Table 1). All participants experienced an increase in fitness ranging from ~3% to ~29%.

For rest and active MEP RCs, paired t‐tests revealed no significant differences in AUCs before versus after 6 weeks of HIIT (Table 1), suggesting that the exercise protocol, despite inducing gains in aerobic fitness, did not lead to changes in corticospinal excitability in sedentary males.

**Figure 4:**
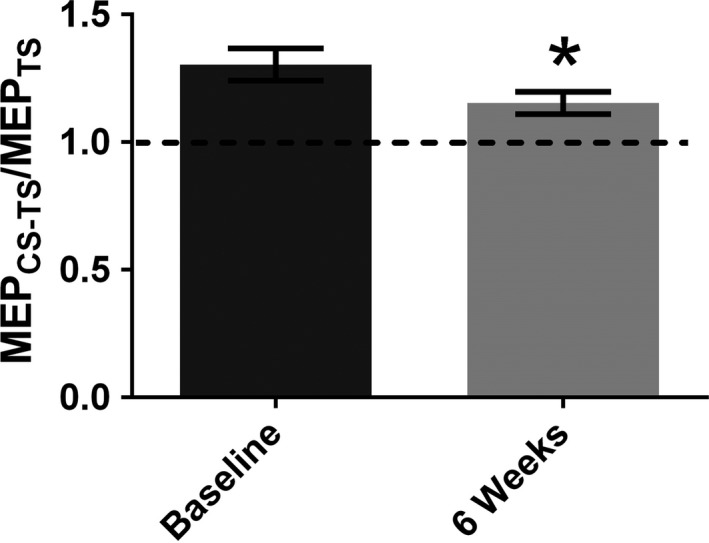
Intracortical facilitation. Group‐averaged intracortical facilitation (ICF) (with standard error) for all participants (*n* = 18), displaying significantly decreased ICF in sedentary males after 6 weeks of 5‐by‐1‐min HIIT. * indicates significance of *P* < 0.05.

ICF was significantly reduced (Fig. 4, Table 1), while SICI showed no change (Table 1) following the 6‐week HIIT intervention.

No significant changes in serum BDNF, IGF‐1, total CTSB, or pro‐CTSB were observed in sedentary males (Table 1).

In summary, these data revealed that 6 weeks of 5‐by‐1‐min HIIT significantly increased aerobic capacity in sedentary males. Further, gains in fitness were accompanied by a reduction in ICF but did not influence corticospinal excitability, SICI or serum levels of BDNF, IGF‐1, total CTSB, and pro‐CTSB.

**Figure 5:**
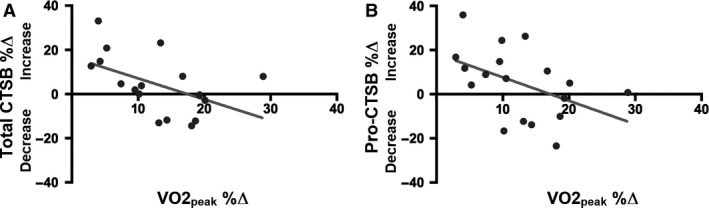
Hierarchical linear regression analysis model 1. A negative relationship between percent change in VO2_peak_ and percent changes in serum levels of total CTSB (A) and pro‐CTSB (B) was found. Results indicate that larger VO2_peak_ gains are associated with decreases in total and pro‐CTSB.

Model 1 of the hierarchical linear regression analysis showed a relationship between VO2_peak_ %Δ and percent changes in serum levels of total CTSB (*β* = −0.976, *P* = 0.051; Fig. 5A, Table 2) and pro‐CTSB (*β* = −1.136, *P* = 0.047; Fig. 5B, Table 2) such that larger gains in aerobic capacity were associated with decreases in peripheral total and pro‐CTSB. No relationship between VO2_peak_ %Δ and percent changes in AUC_Rest_ %Δ, AUC_Active_ %Δ, ICF %Δ, SICI %Δ, BDNF %Δ, IGF‐1%Δ, and OSPAN %Δ was found (Table 2).

**Table 2:** Hierarchical linear regressions

Dependent Measures		*β*	SE	*P*‐value	95% CI	*R* ^2^	Adjusted *R* ^2^	Δ*R* ^2^
AUC_Rest_	Model 1					0.094	−0.027	
	Age	4.026	3.349	0.248	97.5			
	VO2_peak_	−0.884	1.753	0.621	2.5			
	Model 2					0.161	−0.049	−0.067
	Age	4.075	3.858	0.312	97.5			
	VO2_peak_	0.626	2.441	0.802	2.5			
	VO2_peak_ × BDNF Val66Met	−2.019	1.897	0.308				
AUC_Active_	Model 1					0.000	−0.142	
	Age	−0.142	1.834	0.940	97.5			
	VO2_peak_	−0.012	0.960	0.990	2.5			
	Model 2					0.022	−0.245	−0.022
	Age	0.705	2.340	0.769	97.5			
	VO2_peak_	−0.551	1.718	0.754	2.5			
	VO2_peak_ × BDNF Val66Met	0.363	1.375	0.797				
ICF	Model 1					0.105	−0.022	
	Age	−0.922	1.169	0.444	97.5			
	VO2_peak_	−0.735	0.744	0.340	2.5			
	Model 2					0.174	−0.051	−0.069
	Age	−0.317	1.313	0.814	97.5			
	VO2_peak_	0.475	0.862	0.592	2.5			
	VO2_peak_ × BDNF Val66Met	−0.608	0.671	0.385				
SICI	Model 1					0.012	−0.129	
	Age	1.374	3.473	0.698	97.5			
	VO2_peak_	0.090	1.821	0.961	2.5			
	Model 2					0.535	0.408	−0.523
	Age	3.833	2.597	0.168	97.5			
	VO2_peak_	−3.446	1.649	0.061	2.5			
	VO2_peak_ × BDNF Val66Met	4.050	1.299	**0.010**				
BDNF	Model 1					0.198	0.091	
	Age	2.095	4.020	0.610	97.5			
	VO2_peak_	−4.015	2.104	0.076	2.5			
	Model 2					0.215	0.018	−0.017
	Age	0.712	4.852	0.886	97.5			
	VO2_peak_	−5.339	3.070	0.108	2.5			
	VO2_peak_ × BDNF Val66Met	1.910	2.386	0.439				
IGF‐1	Model 1					0.071	−0.053	
	Age	0.485	0.742	0.524	97.5			
	VO2_peak_	−0.362	0.388	0.367	2.5			
	Model 2					0.142	−0.072	−0.072
	Age	0.851	0.894	0.360	97.5			
	VO2_peak_	−0.634	0.566	0.284	2.5			
	VO2_peak_ × BDNF Val66Met	0.230	0.440	0.610				
Total CTSB	Model 1					0.248	0.140	
	Age	0.226	0.954	0.816	97.5			
	VO2_peak_	−0.976	0.457	**0.051**	2.5			
	Model 2					0.273	0.092	−0.026
	Age	0.093	1.030	0.930	97.5			
	VO2_peak_	−1.210	0.652	0.088	2.5			
	VO2_peak_ × BDNF Vall66Met	0.256	0.506	0.622				
Pro‐CTSB	Model 1					0.254	0.154	
	Age	0.963	1.003	0.352	97.5			
	VO2_peak_	−1.136	0.525	**0.047**	2.5			
	Model 2					0.269	0.086	−0.015
	Age	0.237	1.130	0.837	97.5			
	VO2_peak_	−0.986	0.715	0.193	2.5			
	VO2_peak_ × BDNF Val66Met	−0.134	0.556	0.813				
OSPAN	Model 1					0.028	−0.148	
	Age	−0.483	0.901	0.603	97.5			
	VO2_peak_	0.097	0.482	0.844	2.5			
	Model 2					0.198	−0.103	−0.169
	Age	−0.598	1.106	0.604	97.5			
	VO2_peak_	0.641	0.687	0.378	2.5			
	VO2_peak_ × BDNF Val66Met	−0.632	0.522	0.260				

AUC, area under the curve; BDNF, brain‐derived neurotrophic factor; CI, confidence interval; CTSB: cathepsin B; ICF, intracortical facilitation; IGF‐1, insulin‐like growth factor 1; OSPAN, operation span task; SE, standard error; SICI, short‐interval intracortical inhibition; VO2_peak_, peak oxygen uptake.

**Figure 6:**
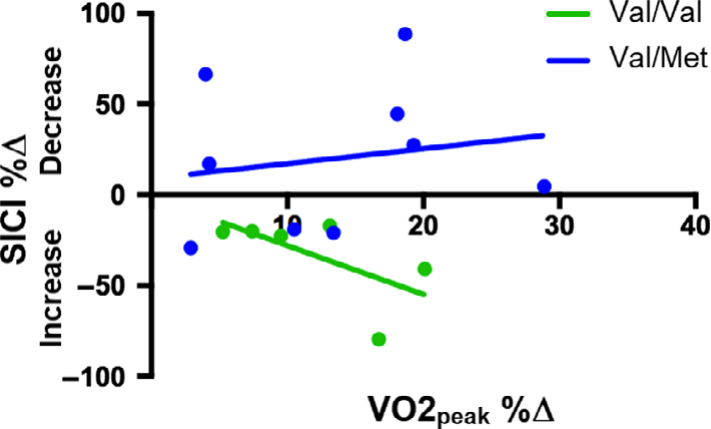
Relationship between percent change in VO2_peak_ and percent change in SICI. A significant interaction between percent change in VO2_peak_ and BDNF Val66Met polymorphism was revealed for SICI. Only Val/Val carriers showed a significant relationship between percent change in VO2_peak_ and percent change in SICI such that greater gains in VO2_peak_ were associated with greater increases in the depth of SICI.

Model 2 revealed a significant VO2_peak_ %Δ X BDNF Val66Met interaction (*β*=4.050, *P* = 0.010; Table 2) for SICI %Δ. Subsequent post hoc analyses indicated that only Val/Val carriers demonstrated a significant relationship between changes in VO2_peak_ and changes in SICI (*β* = −4.250, *P* = 0.049; Fig. 6) such that greater gains in CRF were associated with larger increases in SICI (i.e., greater GABA_A_‐mediated intracortical inhibition). No interaction was found between VO2_peak_ %Δ X BDNF Val66Met for AUC_Rest_ %Δ, AUC_Active_ %Δ, ICF %Δ, BDNF %Δ, IGF‐1%Δ, total CTSB %Δ, pro‐CTSB %Δ, and OSPAN %Δ (Table 2).

## Discussion

While there is evidence that demonstrates changes in M1 excitability following a single bout of aerobic exercise (Takahashi et al. 2011; Singh et al. 2014a, 2014b; Lulic et al. 2017), there are no reports of the effects of long‐term exercise interventions on corticospinal output and intracortical circuitry. In the present study, we examined whether increases in CRF following a 6‐week HIIT protocol at work‐loads of ~105–135% VO2_peak_ were accompanied by changes in corticospinal excitability, TMS‐evoked circuits, and WM in sedentary, healthy males. Further, we investigated whether serum levels of BDNF, IGF‐1, and CTSB, which are thought to contribute to exercise‐induced changes in brain plasticity and memory, were elevated after this exercise protocol. We found that the HIIT intervention resulted in a robust gain in CRF after 6 weeks. We also demonstrated that the increase in fitness was not paralleled by changes in AUC_Rest_, AUC_Active_, SICI, serum levels of BDNF, IGF‐1, total CTSB, pro‐CTSB, or WM capacity. However, we observed a reduction in ICF following the exercise intervention. Further, we determined an association between fitness gains and total and pro‐CTSB such that greater improvements in aerobic capacity were associated with decreases in total and pro‐CTSB. Lastly, we found that only in Val/Val carriers greater fitness gains were associated with larger increases in SICI.

In line with Phillips et al. (2017), we showed that five high‐intensity, 1‐min bouts of exercise were effective in increasing aerobic capacity in sedentary males after 6 weeks, confirming that this time‐efficient exercise protocol is efficacious in yielding improvements in fitness and could be an alternative to high‐volume training in rehabilitative settings.

Previous studies have demonstrated that a single session of aerobic exercise modulates M1 excitability, suggesting that acute exercise might promote short‐term plasticity within M1 (Takahashi et al. 2011; Singh et al. 2014a, 2014b; Lulic et al. 2017). We report that 6 weeks of HIIT, contrary to a single bout, was not accompanied by changes in corticospinal excitability or the TMS‐evoked circuit SICI, indicating that the propensity for long‐term M1 plasticity was unaltered after the HIIT intervention. Interestingly, we further observed that Val/Val carriers who experienced greater gains in fitness showed larger increases in SICI, suggesting that the BDNF Val/Val genotype might moderate the relationship between CRF and GABA_A_‐mediated intracortical inhibition. This is consistent with previous studies reporting that physical activity is correlated with greater hippocampal and temporal lobe volumes and better episodic memory only in Val/Val individuals (Brown et al. 2014; Canivet et al. 2015). Further, Nascimento et al. (2015) found that only Val/Val homozygotes displayed increased plasma BDNF after a 16‐week multimodal exercise program, while Keyan and Bryant (2017) showed that only Val/Val carriers had strong emotional memory formation following 10 min of intense exercise. These findings suggest that the Val/Val genotype might be involved in modulating the effects of exercise on brain plasticity, structure, and function and that individualized programs might be important to maximize the beneficial effects of exercise.

The present work also investigated intracortical excitation within M1 and showed that, similar to acute exercise (Lulic et al. 2017), ICF was reduced after 6 weeks of HIIT in sedentary males. Our finding suggests that acute and chronic exercise might have comparable effects on ICF modulation. However, Singh et al. (2014a, 2014b) reported both an increase and no change in ICF following a single bout of moderate‐intensity lower‐limb exercise. Presently, it cannot be ruled out whether long‐term and acute exercise protocols have a similar influence on cortical excitation. ICF is thought to reflect activation of glutamatergic interneurons and N‐methyl_D_‐aspartate (NMDA) receptors (Liepert et al. 1997; Ziemann et al. 1998). Suppression of ICF following long‐term exercise might help maintain excitability within a physiological range and prime the release of GABAergic inhibition (i.e., decrease in SICI) immediately after a single bout of exercise (Singh et al. 2014a, 2014b; Smith et al. 2014; Lulic et al. 2017). The acute reduction in SICI might promote neuroplasticity such as early acquisition and consolidation of motor skills facilitating motor learning. This can lead to improved performance and recovery in rehabilitative settings as supported by poststroke studies (Stinear et al. 2008; Blicher et al. 2009).

BDNF and IGF‐1 are among the factors that have been shown to modulate exercise‐induced brain plasticity, resulting in improvements in cognition and memory (Carro et al. 2000; Trejo et al. 2001; Gómez‐Pinilla et al. 2002; Ding et al. 2006; Stranahan et al. 2009; Bechara and Kelly 2013). Increases in peripheral BDNF have been repeatedly reported after a single bout of aerobic exercise (Gold et al. 2003; Ferris et al. 2007; Tang et al. 2008; Bos et al. 2011; Griffin et al. 2011; Cho et al. 2012; Nofuji et al. 2012; Schmolesky et al. 2013; Mang et al. 2014; Skriver et al. 2014). However, no changes in serum BDNF or IGF‐1 have been observed following long‐term aerobic exercise (Voss et al. 2013; Maass et al. 2016; Heisz et al. 2017; Gourgouvelis et al. 2018). Consistently, we demonstrated that 6 weeks of HIIT induced gains in aerobic capacity but left serum levels of BDNF and IGF‐1 in sedentary males unaltered. As shown by Leckie et al. (2014), it is possible that long‐term exercise protocols induce significant changes in peripheral BDNF only in older individuals (≥65 years). Further, although 6 weeks of HIIT did not change serum levels of BDNF and IGF‐1, it might facilitate increases in peripheral BDNF and IGF‐1 immediately after a single exercise bout.

In the present study, we also assessed serum levels of the lysosomal cysteine protease, cathepsin B. Moon et al. (2016) reported that plasma levels of CTSB were elevated in young adults after 4 months of aerobic exercise and that CTSB increases were associated with gains in CRF and hippocampal‐dependent memory. Contrary to Moon et al. (2016) but similar to Gourgouvelis et al. (2018), who assessed plasma CTSB in sedentary young adults after 8 weeks of moderate to vigorous aerobic exercise, we found no changes in serum total or pro‐CTSB levels following 6 weeks of HIIT. However, we observed that larger gains in aerobic capacity were associated with decreases in serum levels of total and pro‐CTSB. It is possible that the decreases in serum CTSB associated with the greatest fitness gains might reflect a higher demand of enzymatically active CTSB (mature form) in response to exercise requiring increased cleavage of the inactive CTSB precursor (pro‐CTSB) into the active, mature CTSB (Mach et al. 1994; Mort and Buttle 1997; Hook et al. 2015). Interestingly, Moon et al. (2016) proposed that, following exercise, CTSB is secreted by skeletal muscles and, being able to cross the blood–brain barrier, increases BDNF which in turn promotes brain plasticity, ultimately improving cognition and memory function.

Exercise has beneficial effects on memory and executive function (Cotman and Berchtold 2002; Colcombe and Kramer 2003; Hillman et al. 2003; Chang et al. 2014). WM is an important aspect of executive function involved in temporarily storing, maintaining, and updating information for the execution of high‐order cognitive processes such as learning and reasoning (Engle 2002; Baddeley 2003). Few studies have investigated whether exercise influences WM, and results are inconsistent. Coles and Tomporowski (2008) as well as Li et al. (2014) observed no effect of aerobic exercise on WM. Conversely, McMorris et al. (2011) found that acute, moderate‐intensity exercise improves speed but not accuracy in WM, while Sibley and Beilock (2007) showed that acute exercise benefits WM only in individuals with poor WM. Further, both Pontifex et al. (2009) and Martins et al. (2013) reported that acute aerobic exercise, but not resistance exercise (Pontifex et al. 2009), positively influences WM. Lastly, Gourgouvelis et al. (2018), who assessed executive function, learning and memory using the Cambridge Neuropsychological Test Automated Battery (CANTAB), showed that 8 weeks of moderate‐to‐vigorous aerobic exercise did not change cognitive performance in sedentary, young adults. In the current work, we showed that WM capacity was unaltered after 6 weeks of HIIT in sedentary males. It is possible, as suggested by Li et al. (2014), that, even though the HIIT intervention did not have a measurable effect on OSPAN performance, it might have nonetheless had an impact on the activity of the brain regions involved in modulating WM.

It is interesting to note the similarities in outcome between Gourgouvelis et al. (2018), who also tested sedentary individuals, and the present work. Indeed, neither study demonstrated an effect of exercise training on BDNF, CTSB, or WM. Gourgouvelis et al. (2018) used a combination of resistance and moderate‐to‐vigorous‐intensity aerobic training for 8 weeks, while we used a 5‐by‐1‐min HIIT protocol for 6 weeks. Taken together, these studies suggest a resistance to change in response to exercise in sedentary individuals. It remains unknown whether this is a characteristic of sedentary individuals or whether longer and more intense training interventions are needed to alter neurophysiological and biochemical measures in sedentary individuals.

### Limitations

Despite our finding of reduced ICF following the HIIT intervention, we cannot conclude that ICF is a mediator of exercise‐induced neuroplasticity. This should be investigated in future research using a two‐armed design with a control group not experiencing HIIT. Since peripheral BDNF levels fluctuate during the menstrual cycle (e.g., higher in the luteal *vs*. the follicular phase) (Begliuomini et al. 2007; Pluchino et al. 2009; Cubeddu et al. 2011), we did not examine females in this study. There is evidence that females and males respond differently to exercise, for example, females show lower total carbohydrate oxidation and serum leptin than males after the same training protocol (Tarnopolsky et al. 1990, 1995; Hickey et al. 1997; Friedlander et al. 1998). Thus, it should be addressed in future research whether 6 weeks of HIIT influence TMS and serum measures in sedentary females. Further, we did not examine whether short‐term (i.e., immediately after a single exercise session) neuroplasticity is increased following six consecutive weeks of HIIT. It is also possible that extending the duration of the exercise protocol (e.g., 1 year) might lead to significant changes in corticospinal excitability, SICI and serum factors as well as increases in WM capacity. In addition, only young adults were tested here, and thus, it remains to be established whether 6 weeks of HIIT have a higher impact on facilitating motor cortex excitability and on priming neuroplasticity in older adults than in younger adults. Lastly, due to our limited sample size (*n* = 2, Met/Met), we were not able to determine whether the Met allele constitutes a disadvantage for achieving exercise‐induced benefits.

## Conclusions

Our findings confirm that the time‐efficient HIIT protocol robustly increases CRF and demonstrate that this increase is not accompanied by changes in corticospinal excitability, serum biochemical markers, and WM. Understanding how aerobic exercise modulates neural activity within M1 has implications for the use of exercise as an intervention to modify M1 neural activity, for example, following neurological injury. The literature thus far suggests that exercise does not influence BDNF, CTSB, or WM in sedentary individuals.

## Conflict of Interest

All authors have no potential sources of conflict of interest to declare.
